# Influence of Biopolymer–Biopolymer Interactions on Selected Rheological Properties of Aqueous Ionic Hydrocolloid Solutions

**DOI:** 10.3390/molecules30122482

**Published:** 2025-06-06

**Authors:** Joanna Kruk, Kacper Kaczmarczyk, Paweł Ptaszek, Anna Ptaszek

**Affiliations:** 1 Department of Engineering and Machinery for Food Industry, Faculty of Food Technology, University of Agriculture in Krakow, Balicka 122, 30-149 Krakow, Poland; pawel.ptaszek@urk.edu.pl; 2 Department of Chemistry, Faculty of Food Technology, University of Agriculture in Krakow, Balicka 122, 30-149 Krakow, Poland; kacper.kaczmarczyk@urk.edu.pl; 3 Centre of Innovation and Research on Prohealthy and Safe Food, University of Agriculture in Krakow, Balicka 104, 30-149 Krakow, Poland; anna.ptaszek@urk.edu.pl

**Keywords:** shear viscosity, extensional viscosity, normal force, structural properties, time constants

## Abstract

The influence of biopolymer–biopolymer chain interactions on selected rheological properties of aqueous solutions from konjac (KG), xanthan gum (XG), and carboxymethyl cellulose (CMC) was investigated using viscosity measurements in extensional and shear flow, as well as normal force ($F_{N}$) measurements generated in shear flow. It was found that a KG solution of 0.05% behaves as a Newtonian fluid. Other solutions of KG (0.1, 0.2%), XG, and CMC revealed a non-linear dependence of viscosity on the shear rate. The extensional viscosity dependence on the elongation rate was non-linear and indicated shear-thinning over the entire KG concentration range, with the lowest values noted at 0.05% (0.5–0.8 Pas) and the highest at 0.2% (1.0–1.3 Pas). Similar observations were obtained with 0.1% XG and CMC solutions. Analysis regarding the shear rate dependence of the $F_{N}$ showed that hysteresis was observed for all KG concentrations tested. Only for the 0.2% KG solution were the $F_{N}$ values negative over the entire range of shear rates estimated, as in the case of the XG and CMC solutions. The obtained time constants from the DeKee model indicate the dominance of elastic contributions for the XG and CMC solutions and viscous contributions for the CMC solutions in the case of an extensional flow.

## 1. Introduction

Polysaccharides are natural polymers (biopolymers) produced by plants, microorganisms, and animals. They are characterised by a complex chain structure, which is composed of monosaccharides such as glucose, mannose, or galactose linked by glycosidic bonds. Type 1–4 bonds build the linear part of the chain, while 1–6 bonds are responsible for the formation of side branches with different molecular characteristics. Numerous functional groups such as hydroxyl, amide, carboxyl, or acetyl may be present in the chain, which give individual character to the polysaccharides and are responsible for their unique properties in aqueous solutions [1,2,3,4,5]. The presence and nature of branching chains, as well as the concentration of the macromolecule length, decide the interactions in water [3,4,5]. As a result of the biopolymer–solvent and biopolymer–biopolymer interactions, three characteristic regimes of the concentration can be observed: diluted, semi-diluted, and concentrated, which are delineated by two critical concentrations: c* (first critical concentration) and c** (second critical concentration) [6,7,8]. These values can be determined with the aid of various measurement techniques, including SLS (Static Light Scattering), DLS (Dynamic Light Scattering), capillary viscometry, and rotational rheometry. Knowledge of the interactions between polysaccharides and water as a solvent is used in practice to shape selected functional properties (among others, rheological, textural, and water-binding capacities) of many food products. Polysaccharides are thus used as thickeners, gelling agents, emulsifiers, and anti-syneresis substances. The group of polysaccharides commonly used as food additives includes xanthan gum (E415), konjac gum (E425), and a cellulose derivative: cellulose gum (carboxymethyl cellulose E466). These three hydrocolloids belong to the group of ionic polysaccharides and their rheological properties in shear and extensional flow are still being intensively studied [5,9].

Xanthan gum (XG) is an exopolysaccharide industrially produced by Xanthomonas campestris bacteria through the fermentation of carbohydrates. The main chain of this heteropolysaccharide is composed of glucose and trisaccharide side chains consisting of glucuronic acid and mannose at a 2:2:1 ratio [10]. Xanthan gum solutions are among the most extensively studied aqueous polysaccharide systems. In the case of XG diluents, which are branched polysaccharides, critical concentrations as a function of weight-average molecular weight have been determined (values are summarised in Table 1) [6,11,12,13,14], and the effect of concentration on the rheological properties of aqueous XG systems has also been studied, including ionic strength solutions [6,14,15]. The effect of XG concentration on the normal force generated during a shear flow and extensional viscosity (using the opposite nozzle, a stagnant flow, or capillary outflow (filaments), has been studied [14,16,17,18,19].

Konjac gum (KG) is a neutral polysaccharide obtained from the tubers of *Amorphophallus konjac*. Chains are formed by mannose and glucose at a 1.6:2 ratio [20]. The molecular structure of KG determines its properties in aqueous solutions, shaping the critical concentration values and quality of biopolymer–solvent interactions [2,4,7,21,22,23,24] as well as rheological properties [4,7,21,22,24].

Carboxymethyl cellulose (CMC) belongs to cellulose derivatives and is a half-synthetic anionic polysaccharide characterised by a linear chain structure formed of glucose units with partial substitution by the carboxymethyl group. An average number of carboxymethyl groups repeating unit of CMC is in the range 0.4–1.5 [25]. As with other polysaccharide hydrocolloids, critical concentration values have been determined as a function of weight-average molecular weight [8,26,27,28]. The rheological properties of CMC solutions in shear flow are well known [3,26,28,29,30,31], while there is increasing work being carried out on the properties of solutions in an extensional flow [18,32,33].

The aim of this study was to investigate the influence of biopolymer concentrations on selected rheological properties of structured fluids (aqueous solutions of konjac gum, xanthan gum, and carboxymethyl cellulose) using viscosity measurements in extensional and shear flow, as well as estimates of the normal force generated in shear flow, in light of biopolymer–biopolymer or biopolymer–water interactions. In addition, an attempt was made to use the De Kee and combined De Kee and Papanastasiou model to describe the rheological properties in both extensional and shear flow.

**Table 1:** Structural properties of KG, XG, CMC chains: weight-average molecular weight (M_w_), average osmotic molar mass (M_osm_), second virial coefficient (A_2_), first critical concentration (c*), second critical concentration (c**). Time constants from rheological models (De Kee, combination of De Kee and Papanastasiou models)—t_1_, t_2_, for shear and elongational flow. Bold values of time constants indicate their dominant influence in the formation of rheological properties.

	M_w_, kg/mol	M_osm_, kg/mol	A_2_ (30 °C) mol mL g^−2^	Mw, kg/mol	c*, %	c**, %	c, %	Elong			Shear				
											Up		Down		
								t_1_, ms	t_2_, ms	χ ^2^	t_1_, ms	t_2_, ms	t_1_, ms	t_2_, ms	χ ^2^
KG	990	1557	−2.13 × 10^−4 (a)^	1030 ^(1)^	0.033	0.072	c*~0.05	1	50		Newtonian viscosity 2.35 mPas				
							c**~0.10 ^eq.4^	2	50	10^−3^	14	1000	10	350	10^−6^
							c** < 0.20	2	50	10^−5^	6	**150**	**5**	100	10^−6^
CMC	300	2570	9.84 × 10^−5 (b)^	280 ^(2)^	0.02	0.1	c**~0.10	**3**	300	10^−5^	5	**200**	**4**	100	10^−6^
XG	1960	-	−1.36 × 10^−4 (b)^	2160 ^(3)^	0.023	0.07	c** < 0.10	**5**	100	10^−4^	1	**200**	9	**200**	10^−6^

^(a)^ [23], ^(b)^ [34], ^(1)^ [7], ^(2)^ [8], ^(3)^ [6]; ^eq.4^ combination of De Kee and Papanastasiou models.

## 2. Results

### 2.1. Effect of Konjac Gum (KG) Concentration on Rheological Properties

**Figure 1:**
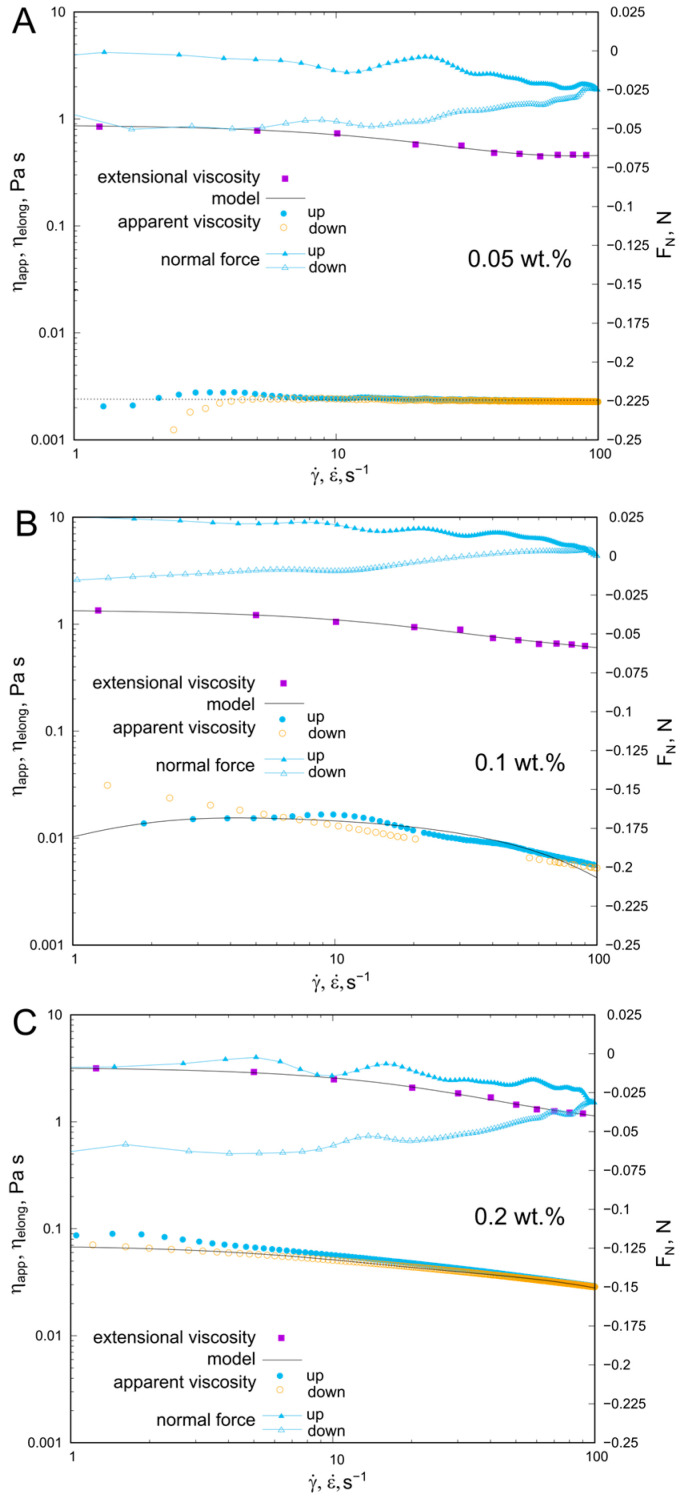
Effect of KG concentration on rheological properties: dependence of apparent viscosity ($\eta_{app}$), normal force $\left(F_{n}\right)$ on shear rate ($\dot{\gamma }$) and extensional viscosity ($\eta_{elong})$ on elongation rate ($\dot{\varepsilon })$ at 25 °C. (**A**) 0.05 wt.% KG solution, (**B**) 0.1 wt.% KG solution, (**C**) 0.2 wt.% KG solution. Solid black lines represent De Kee or combined De Kee-Papanastasiou model fitted to the experimental data.

The effect of polysaccharide concentration on the rheological properties is illustrated in Figure 1. At the lowest concentration (c = 0.05%~c*), the system was found to behave as a Newtonian fluid, and its viscosity was 2.35 mPas. Some divergence in the $\eta \left(\dot{\gamma }\right)$ relationship was observed at the lowest shear rate conditions (<4 s^−1^).

The dependence of extensional viscosity on elongation rate was non-linear and indicated extensional thinning. The $\eta_{elong}$ values were at least 10 times the Newtonian viscosity and varied between 0.3 Pas and 1 Pas. The dependence of normal force values on shear rate indicates a hysteresis of $F_{N}$ changes depending on the direction of shear rate development. In conditions of an increasing shear rate, values of $F_{N}$ > 0 were in the range of up to ~50 s^−1^. In the second part of the experiment (decreasing shear rate), the course of $F_{N}$ differed from the previous one, and values of $F_{N}$ < 0 were above the entire shear rate range. A twofold increase in KG concentration (c = 0.1%~c**) caused clear changes in the rheological behaviour of the solution. First of all, a non-linear dependence of viscosity on shear rate was found over the entire $\dot{\gamma }$ range investigated, as well as evident hysteresis of the viscosity curves. In addition, it was observed that the nature of the viscosity hysteresis loop changes with an increasing shear rate, in the range of shear rates up to 7 s^−1^. The apparent viscosity measured in conditions of a decreasing shear rate (‘down’) is higher, while under conditions of 7 < $\dot{\gamma }$ < 20 s^−1^ it was lower than $\eta_{app}$ determined during the first stage of the experiment (‘up’ increasing shear rate). Furthermore, it was found that in the range of lower shear rates, the apparent viscosity increases with the shear rate. Analysis of the $F_{N}\left(\dot{\gamma }\right)$ relationship demonstrated that, under increasing shear rate conditions, $F_{N}$ > 0 was in the full $\dot{\gamma }$ range, while in the second part of the experiment, above 60 s^−1^ $F_{N}$ < 0. The extensional viscosity decreased non-linearly with increasing elongation rate, and its values were higher than in the 0.05 wt.% concentration. For the solution with the highest concentration (c = 0.2% > c**), a non-linear dependence of viscosity on shear rate and extensional viscosity on elongation rate was found. Elongational apparent viscosity assumed the highest values, decreasing with $\dot{\varepsilon }$ from 3 Pas to 1 Pas. Apparent viscosity decreased from 0.1 Pas to 0.03 Pas, and hysteresis of the shear rate dependence of viscosity was observed in both stages of the rheological experiment. For this solution, $\eta_{app}$ − ‘up’ had higher values than $\eta_{app}$ − ’down’ throughout the gamma range. The course of the $F_{N}$ dependence ($\dot{\gamma }$) was characterised by hysteresis. However, it was above the entire shear rate range of $F_{N}$ < 0.

### 2.2. Behavior of XG and CMC Concentrated Solutions

**Figure 2:**
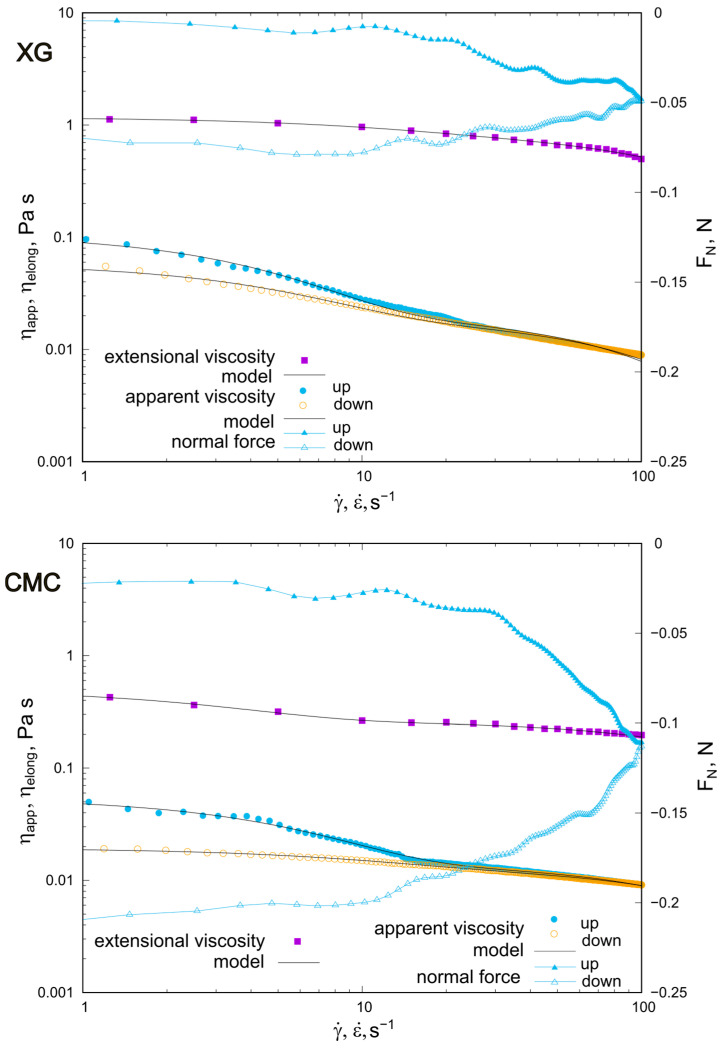
Rheological properties of 0.1% XG and CMC solutions: dependence of apparent viscosity ($\eta_{app}$), normal force $\left(F_{n}\right)$ on shear rate ($\dot{\gamma }$) and extensional viscosity ($\eta_{elong})$ with regard to elongation rate ($\dot{\varepsilon })$ at 25 °C. Solid black lines represent the De Kee model fitted to the experimental data.

The rheological properties of XG and CMC solutions with a concentration of 0.1% are shown in Figure 2. For both polysaccharides, the chosen concentration is close to the second critical concentration (~c**). The XG solution shows shear-thinning and hysteresis of the viscosity curves. The largest differences in the $\eta_{app}\left(\dot{\gamma }\right)$ relationship were found in the range of lowest shear rates (<10 s^−1^), for which the largest differences in normal force ($F_{N}$) were also recorded. The relationship of $F_{N}\left(\dot{\gamma }\right)$ for the test stage corresponding to increasing shear rate is decreasing, with values of $F_{N}$ < 0 and varying from 0 N to −0.05 N. In the second stage of the hysteresis loop experiment (decreasing shear rate values), the value of the normal force remains negative and increases in absolute value. Again, the hysteresis phenomenon is observed, with the greatest intensity in the lowest shear rate range. The values of the extensional viscosity are more than 10 times higher than the apparent viscosity and decrease non-linearly with the elongation rate. The greatest hysteresis in the course of the $F_{N}\left(\dot{\gamma }\right)$ relationship was observed for the CMC solution, and for this system, the normal force, in terms of absolute value, was the highest: in conditions of an increasing shear rate, it decreased from −0.025 N to −0.11 N, and in the second part of the experiment (‘up’), with a decreasing $\dot{\gamma }$ value, it decreased to 0.21 N. The dependence of extensional viscosity on elongation rate and apparent viscosity on shear rate was non-linear. For the $\eta_{app}\left(\dot{\gamma }\right)$ run, hysteresis was found in the shear rate range up to 10 s^−1^.

### 2.3. Effect of Concentration on Characteristic Time Constants

Modelling of the rheological properties concerning the studied systems was carried out using an exponential rheological model. Rheological characteristic times from the DeKee equation describing shear-thinning systems (analogy to relaxation times from Maxwell’s model) with lower values can be interpreted as typical of systems capable of storing mechanical energy. The smaller the value of this parameter, the closer the behaviour is to elasticity. Two characteristic times were determined for all the systems studied, and the pre-exponential coefficients ($\eta_{1}$, $\eta_{2}$) were interpreted as a kind of ‘intensity’ for a given rheological characteristic time. The results are shown in Table 1.

In the case of shear flow, estimation of DeKee model parameters was carried out for viscosity curves determined in conditions of an increasing (up) and decreasing (down) the shear rate. The rheological behaviour of XG solutions (0.1 wt.%) is dominated by the viscosity mechanism, as evidenced by the dominant, larger than $t_{1}$ values of the second characteristic $t_{2}$ time. This behaviour is evident on both the ‘up’ and ‘down’ curves for the XG solution. The solution of 0.1 wt.% KG shows a more complex rheological behaviour. Above all, the values of the characteristic $t_{2}$ times are much higher than the analogous ones estimated for the XG and CMC solutions, indicating a clear contribution of viscosity contributions in shaping the rheological properties (values of $\eta_{1}$~$\eta_{2}$). In Table 1, the effect is shown of KG concentration on the values of the DeKee rheological equation parameters. The 0.05% solution (c~c*) behaves as a Newtonian system. The highest values of $t_{1}$ and $t_{2}$ correspond to a concentration close to the second critical concentration, while for c = 0.20%, they decrease to values close to those estimated for XG and CMC. For the 0.10% solution, the combined De Kee and Papanastasiou model was fitted to the experimental data due to the maximum apparent viscosity dependence on the shear rate.

In the case of extensional flow, a dominance of shorter characteristic times was found, which, according to the interpretation given earlier, can be regarded as the dominance of elastic effects in shaping rheological properties. For XG and CMC solutions, the values of the second characteristic time are much larger than $t_{1}$, but the values of the pre-exponential coefficient from the DeKee equation indicate a small contribution of viscous phenomena to the image of rheological behaviour in extensional flow. A slightly different situation was observed in KG solutions, for which the estimated values of the second characteristic time are smaller than for XG and CMC and did not depend on the concentration of this hydrocolloid. Moreover the values $\eta_{2}$~$\eta_{1}$, highlighted the influence of viscosity contributions on the properties of these solutions.

## 3. Discussion

The rheological behaviour of the systems under study can be analysed in light of structural properties, which are determined by the structure of the polysaccharides and the interaction with water (solvent) or those between these chains. In addition to temperature and solvent quality, the biopolymer concentration and weight-average molecular weight determine the quality of these interactions. The examined polysaccharides belong to the ionic group, with xanthan gum showing a branched-chain structure and the highest weight-average molecular weight (Table 1). In the evaluated concentration region (c~c*), there is a conformational change from the diluted characteristic (spherical arrangement clusters) to an ordered structure, including the possible formation of junction zones between xanthan gum chains [6,11]. Due to the negative value of the second virial coefficient (Table 1), this can be interpreted as a kind of ‘aggregation’ regarding the XG chains and consequent phase separation. The concentrated-junction zones phase is characterised by a high concentration and a higher degree of order, whereas the dispersed phase is ‘diluted’ and its structure is disordered [6,11].

Similar behaviour is observed for KG solutions, for which water is not a good solvent (Table 1) [1,23]. As indicated by the results, the conformation of KG varies from that characteristic of a statistical cluster to that intermediate between the classical interpenetration of clusters in the semi-diluted region [1] and the formation of entanglement zones characteristic of non-ionic glucomannans (e.g., GG) [7,21,35]. The permeation of CMC chains in a solution depends on the chain length (number of basic units), but due to the affinity of these chains for water ($A_{2}$ > 0), it follows that the mechanism is expected for systems that do not exhibit phase separation built by flexible chains interacting with water through hydrogen bridges [26,30].

All of the solutions studied exhibited complex rheological behaviour manifested by deviation from the exponential model of shear rate stress dependence.

As a result, it was possible to fit the De Kee model or combine the De Kee and Papanastasiou models to the rheological data. Analysis of the coefficient values for shear flow showed some differences between the behaviour of the solutions. In the case of XG solutions, viscosity contributions predominated, demonstrating the irreversible breakdown of the pseudo-gel structure formed by XG chains [36,37,38], as may be evidenced by the dominance of the longer characteristic time determined from the DeKee model. This phenomenon is evident at both stages of the rheological experiment (‘up’ and ‘down’ viscosity curves). Similar behaviour was registered for the CMC solution concerning the flow curve realised at an increasing shear rate, while in decreasing rate conditions, a shorter relaxation time was noted—indicating the predominance of elastic contributions—gaining the advantage. In the case of solutions of both biopolymers, their concentration was close to or above c**, indicating that we are dealing with a fully formed structural fluid. Differences between the behaviour of the structure in the concentrated region are apparent in the realisation of the viscosity curve under a decreasing shear rate: for the XG solution ($A_{2}$ < 0), the mechanism of the system’s response to the applied shear stress does not change, while for CMC ($A_{2}$ > 0), elastic contributions come to the forefront [29].

KG solutions exhibit a wide range of rheological behaviours, from those that are characteristic of Newtonian systems (c~c*), onto the maximum on the flow curve (c~c**) to non-Newtonian shear-thinning. It is noteworthy that under c~c** conditions, once the shear rate of ~7 s^−1^ is exceeded, the apparent viscosity of the ‘down’ stage is clearly higher than the apparent viscosity of the ‘up’ stage. This indicates a clear change in the response mechanism of the tested fluid in reaction to the applied shear stress. This phenomenon is reflected in the combined DeKee and Papanastasiou model adopted, as well as in the values of the characteristic time constants, which indicate the elastic contributions in the increasing shear rate (‘up’) stage. In the second stage of the experiment, the viscous mechanism predominated. A further increase in KG concentration (c > c**) implies the dominance of the viscous mechanism.

Changes in the $F_{N}$ dependence $\left(\dot{\gamma }\right)$ distinguish the behaviours of the CMC solution (c~c**), for which the strongest dependence hysteresis phenomenon was found for increasing and decreasing shear rates. This polysaccharide had the lowest weight-average molecular weight and exhibited $A_{2}$ > 0. The positive value of the second virial coefficient indicates the high affinity of the biopolymer chain for the solvent, which can be interpreted as the ability to bind water in its ‘structure’ [2,29]. This means that CMC chains remain elastic even in c~c** conditions [18,26]. Exceeding c** value results in an enhancement of elastic properties of solutions (visible as an increase in apparent viscosity on the viscosity curve) as manifested by an increase in the value of the characteristic time constant in the Cross model [26]. However, for XG and CMC solutions in the studied concentration range, $F_{N}$ > 0 was not observed. This was most likely due to the fact that it may lead to the formation of a rigid continuous particle network, which can withstand the flow [11,14,18,26,36]. In the case of KG, in c~c** conditions and increasing shear rate, $F_{N}\left(\dot{\gamma }\right)$ > 0 [2] was found. KG chains are not branched similarly to XG. However, in the c~c** range, the generated interactions in the entanglement network are robust enough to produce a flexible structure.

## 4. Materials and Methods

### 4.1. Materials

Commercially available, food-grade powdered gum preparations: konjac (KG), xanthan (XG), and carboxymethyl cellulose (CMC) (Hortimex Sp. z o.o., Konin, Polska) were purchased from local suppliers. Powdered preparations were used to prepare aqueous solutions at concentrations of 0.05, 0.1, and 0.2 wt.% for konjac gum, and 0.1 wt.% for xanthan gum and carboxymethyl cellulose. In order to properly rehydrate the gum preparations, the treatment of the solutions involved two steps. In the first stage, the gums were mixed using magnetic stirrers (500 rpm) for 3 h at ambient temperature (23 °C) In the second stage, the obtained solutions were stored at room temperature for 24 h. After this time, the obtained aqueous gum solutions were subjected to rheological measurements (Section 4.2.1).

### 4.2. Methods

#### 4.2.1. Gel Permeation Chromatography (GPC)

Gel permeation chromatography was used to determine the weight-average molecular weights of KG, XG, and CMC. The chromatography system employed a stationary phase in the form of two polymer-based columns (Ultrahydrogel 2000, Ultrahydrogel 500) (Waters, Milford, MA, USA) connected in a series with an RI detector (Knauer, Berlin, Germany). In the mobile phase, an NaNO_3_ and NaN_3_ solution in water was utilized. This was performed at a concentration of 0.1 mol·L^−1^ and 0.02%, respectively. The flow rate of the mobile phase was set to 0.6 mL·min^−1^, and 100 mL of the sample was injected. The solutions used as samples were prepared using 5 mg of powder in 1 mL of water. The calibration procedure with pullulan standards (Shodex, Tokyo, Japan) was conducted according to the previously described method [39]. All reagents and chemicals were purchased from Sigma-Aldrich (St. Louis, MO, USA).

#### 4.2.2. Extensional Rheology

Extensional flow measurements were conducted with the MTS-02 opposite nozzle device (Roman Pomianowski Laboratory of Electronics, Poznań, Poland), based on the Fuller conception [40], with some alterations. The nozzle’s inner diameter was 2 mm, and the distance between nozzles was set to 1 mm. The examined fluids were sucked out by the nozzles through a high-precision double-syringe pump (New Era Pump Systems Inc., Farmingdale, NY, USA) at various rates, which translated into different extensional rate values. Measurements were carried out at an ambient temperature of 25 (±0.2) °C at selected elongation rate values within the range of 1.3 and 106 s^−1^. The elongation rate was calculated using the following formula:

(1) $$\dot{\varepsilon }=\frac{\frac{\dot{V}}{2}}{A\cdot \frac{d}{2}}$$

where $\dot{\varepsilon }$—elongation (extension) rate value, s^−1^; $\dot{V}$—volumetric flow rate, m^3^·s^−1^; A—area of nozzle openings, m^2^; d—distance between nozzles, m.

Extensional viscosity can be expressed using the formula:

(2) $$\eta_{elong}=\frac{M}{\dot{\varepsilon }\pi R^{2}d}=\frac{F\frac{d}{2}}{\dot{V}}$$

where $\eta_{elong}$ is extensional viscosity, Pa·s, M is rotational torque, N·m; ε is elongation (extension) rate value, s^−1^, R is nozzle radius, m, d is the distance between nozzles, m, F is measured force, N. The experiment was triplicated.

#### 4.2.3. Rotational Rheology

The rheological behaviour of the gum water solutions was characterised using the RS6000 rotational rheometer (ThermoFisher, Karlsruhe, Germany), with cone-plate geometry having the following cone parameters: 60 mm in diameter and 1° angle. Flow curves were determined at 25 °C, with an increasing and decreasing shear rate of 1 to 100 s^−1^ and 100 to 1 s^−1^, respectively—qualitative hysteresis loop test. The duration of each stage, linear increase (‘up’) and decrease (‘down’) of shear rate was 750 s. During the experiment, data on changes in normal force value ($F_{N}$) were also collected. The measurement procedure involved setting the normal force value to zero when the cone sensor reached the position corresponding to the measurement gap. The experiment was triplicated.

#### 4.2.4. Modelling of Rheological Properties

For the obtained rheological data, the values of apparent viscosity ${(\eta }_{app})$ as well as elongational viscosity ${(\eta }_{elong})$ were fitted with the De Kee exponential model, which describes the shear-thinning system [41]. The formula was the following:

(3) $$\eta \left(\dot{\gamma }\right)=\tau_{0}\cdot {\dot{\gamma }}^{-1}+\eta_{1}\cdot \exp\left(-t_{1}\cdot \dot{\gamma }\right)+\eta_{2}\cdot \exp\left(-t_{2}\cdot \dot{\gamma }\right)$$

For 0.1% KG, a combination of De Kee and Papanastasiou models was used [42]:

(4) $$\eta \left(\dot{\gamma }\right)=\tau_{0}\cdot {\dot{\gamma }}^{-1}+\eta_{1}\cdot \exp\left(-t_{1}\cdot \dot{\gamma }\right)+\eta_{2}\cdot \exp\left(-t_{2}\cdot \dot{\gamma }\right)-\eta_{3}\cdot \exp\left(-t_{3}\cdot \dot{\gamma }\right)$$

In both equations, $\tau_{0}$ is the yield stress, Pa, $\dot{\gamma }$ is the shear rate, s^−1^, $\eta_{p}$ is the intensity of the time constant parameter, Pas, and $t_{p}$ is the time constant, s. In the case of applying the presented models to the rheological data obtained during extensional flow, the shear rate $\dot{\gamma }$ is replaced by the elongation rate $\dot{\varepsilon .}$ Interpretation of parameters was conducted on the basis of the available study by Ptaszek [43]. Estimation of both models’ parameters was performed according to the Marquardt–Levenberg method, which was applied as the minimisation algorithm using the least squares method. The target function was defined as follows:

(5) $$\chi_{M-L}^{2}=\sum_{j=1}^{N}{\left[\eta_{j}^{\sigma }-{\hat{\eta }}_{j}\left({\dot{\gamma }}_{j}\right)\right]}^{2}\underset{\tau_{0},\eta_{1},\eta_{2},t_{1},t_{2}}{\to }min$$

where $\eta_{j}^{\sigma }$ are experimental values of apparent or extensional viscosity and ${\hat{\eta }}_{j}\left({\dot{\gamma }}_{j}\right)$ were calculated from the Equations (3) and (4). The value of $\chi^{2}$ indicates the goodness of the De Kee model fit (Table 1).

## 5. Conclusions

The use of viscosity measurements in shear flow, together with normal force acquisition, as well as viscosity in extensional flow, makes it possible to investigate the influence of the interactions occurring in aqueous food gum solutions on their rheological properties. It has been shown that the konjac gum solution, at the lowest concentration, is a Newtonian fluid. Solutions with higher concentrations behave as shear-thinning fluids and exhibit hysteresis of the viscosity curves. Similar observations were made for XG and CMC solutions. The De Kee model and the combined De Kee and Papanastasiou model can be used to describe the rheological properties of aqueous polysaccharide solutions obtained in conditions of both shear and extensional flows. The characteristic times ($t_{1}$,$t_{2}$) determined from the applied models indicate that in the case of the shear flow, with an increasing shear rate, viscous contributions dominate in shaping the rheological properties of all the studied solutions. In contrast, elastic contributors have a predominant influence on the shear flow with a decreasing shear rate (0.2% KG and 0.1% CMC). Extensional flow testing revealed the dominance of elastic effects for XG and CMC solutions. Elastic and viscous effects form the rheological behaviour of KG solutions.

## Data Availability

Data that support the findings of this study are available from the corresponding authors upon reasonable request.
